# Genome Sequence of “*Thalassospira australica*” NP3b2^T^ Isolated from St. Kilda Beach, Tasman Sea

**DOI:** 10.1128/genomeA.01139-14

**Published:** 2014-11-13

**Authors:** Mario López-Pérez, Francisco Rodriguez-Valera, Hayden K. Webb, Russell J. Crawford, Elena P. Ivanova

**Affiliations:** a Universidad Miguel Hernandez, San Juan de Alicante, Spain; b Swinburne University of Technology, Hawthorn, Victoria, Australia

## Abstract

Here, we present the draft genome of “*Thalassospira australica*” NP3b2^T^, a potential poly(ethylene terephthalate) (PET) plastic biodegrader. This genomic information will enhance information on the genetic basis of metabolic pathways for the degradation of PET plastic.

## GENOME ANNOUNCEMENT

Plastic pollution in marine ecosystems is a growing environmental concern. Accumulation of large plastic “islands” in ocean gyres demonstrates the abundance of plastic waste in marine environments (1, 2). Plastic waste has substantial impacts on marine wildlife, posing significant entanglement and ingestion hazards (3, 4) and causing considerable financial costs to the shipping industry (5). Unfortunately, this issue cannot be simply solved. Only a few viable, environmentally friendly disposal methods exist; however, microbial biodegradation represents a promising remediation alternative. The bacterial strain prompting this announcement was originally isolated during research into microorganisms that exhibited potential for the degradation of poly(ethylene terephthalate) (PET). PET is a polymer commonly used in the fabrication of water bottles and many other products.

Gram-negative, aerobic, moderately halophilic gammaproteobacteria with the ability to utilize hydrocarbons as the sole carbon and energy sources were incorporated into the genus *Thalassospira* more than 20 years ago (6). To date, the genus comprises 9 validly named species (7). Currently, there are four *Thalassospira* strains reported to have their full genomes sequenced (8–14). Strain NP3b2^T^ was isolated from an enrichment experiment selecting for strains that degrade poly(ethylene terephthalate) (PET) from seawater collected from the first meter below the water’s surface at St. Kilda Beach, Port Phillip Bay, Tasman Sea, Victoria, Australia (15). The specific location of the studies (GPS coordinates) was 37°51′50″ S 144°58′55″ E. The analyses of the genome of this novel *Thalassospira* species will stimulate further research on the metabolite activity, organic pollutant degradation, physiological and ecological functions, and evolution of the bacteria of the genus *Thalassospira*.

On the basis of the data generated from *in vitro* and *in silico* studies, strain NP3b2^T^ is considered to represent novel species of the genus *Thalassospira*, for which the name “*Thalassospira australica*” NP3b2^T^ is proposed (H. K. Webb, S. H. Nguyen, M. López-Pérez, F. Rodriguez-Valera, R. J. Crawford, and E. P. Ivanova, unpublished data). The genome of strain NP3b2^T^ was sequenced using the IlluminaHiSeq 2000 (100-bp paired-end read) sequencing platform (Macrogen, Korea). The generated reads were trimmed and assembled *de novo* using VELVET, version 0.7.63 (16). The resulting sequence was then submitted to the Microbial Genome Annotation Pipeline (MiGAP) (http://www.migap.org/index.php/en/) (17) and NCBI Prokaryotic Genomes Automatic Annotation Pipeline (PGAAP) for auto-annotation. The open reading frames (ORFs), rRNAs, and tRNAs were also predicted using the MetaGeneAnnotator (MGA) (18), RNAmmer (19), and tRNAscan-SE (20). The size of the draft genome of strain NP3b2^T^ was found to be 4,268,334 bp, comprising 32 contigs, with a G+C content of 53.6%. Strain NP3b2^T^ contained 3,934 predicted genes, 3,875 putative coding sequences (CDS), 5 rRNAs, and 55 tRNAs.

The average nucleotide identity (ANI) (21) and the DNA-DNA hybridization (DDH) values between the draft genome of strain NP3b2^T^ and the four strains already published, *Thalassospira profundimaris* WP0211 (22), *T. xiamenensis* M-5 (23), *T. permensis* NBRC 106175 (24), and *T. lucentensis* QMT2^T^ (25), were 82.2% to 76.6% and 21% to 25.5% (26), respectively, confirming that strain NP3b2^T^ belongs to a new species within the *Thalassospira* genus.

### Nucleotide sequence accession number.

The genome data have been deposited at NCBI under BioProject number PRJNA257045 and accession number JRJE00000000 for “*Thalassospira australica*” NP3b2^T^.
