# Correction for López-Pérez et al., Genome Sequence of “*Thalassospira australica*” NP3b2^T^ Isolated from St. Kilda Beach, Tasman Sea

**DOI:** 10.1128/genomeA.01453-14

**Published:** 2015-01-22

**Authors:** Mario López-Pérez, Francisco Rodriguez-Valera, Hayden K. Webb, Russell J. Crawford, Elena P. Ivanova

**Affiliations:** a Universidad Miguel Hernandez, San Juan de Alicante, Spain; b Swinburne University of Technology, Hawthorn, Victoria, Australia

## AUTHOR CORRECTION

Volume 2, no. 6, e01139-14, 2014. Page 1: The first two sentences of the second paragraph should read as follows. “Gram-negative, aerobic, moderately halophilic alphaproteobacteria with the ability to utilize hydrocarbons as the sole carbon and energy sources were incorporated into the genus *Thalassospira* 12 years ago (6). To date, the genus comprises 8 validly named species (7).”

Page 1: The reference range cited at the end of the third sentence in the second paragraph should be 22 to 25.

Page 2: Reference 6 should be replaced with the following.

6. **López-López A, Pujalte MJ, Benlloch S, Mata-Roig M, Rosselló-Mora R, Garay E, Rodríguez-Valera F**. 2002. *Thalassospira lucentensis* gen. nov., sp. nov., a new marine member of the α-*Proteobacteria*. Int. J. Syst. Evol. Microbiol. **52:**1277–1283. http://dx.doi.org/10.1099/ijs.0.01928-0.

Page 2: References 8 to 14 should be deleted.

